# Preparing for the Worst: Management and Predictive Factors of Open Conversion During Minimally Invasive Renal Tumor Surgery (UroCCR-135 Study)

**DOI:** 10.1016/j.euros.2024.03.009

**Published:** 2024-03-28

**Authors:** Nicolas Branger, Nicolas Doumerc, Thibaut Waeckel, Pierre Bigot, Louis Surlemont, Sophie Knipper, Géraldine Pignot, François Audenet, Frank Bruyère, Alexis Fontenil, Bastien Parier, Cécile Champy, Morgan Rouprêt, Jean-Jacques Patard, François Henon, Gaëlle Fiard, Julien Guillotreau, Jean-Baptiste Beauval, Constance Michel, Simon Bernardeau, Fayek Taha, Richard Mallet, Frederic Panthier, Laurent Guy, Louis Vignot, Zine-Eddine Khene, Jean-Christophe Bernhard

**Affiliations:** a Department of Urology, Institut Paoli Calmettes, Marseille, France; b Department of Urology, CHU Toulouse, Toulouse, France; c Department of Urology, CHU Caen, Caen, France; d Department of Urology, CHU Angers, Angers, France; e Department of Urology, CHU Rouen, Rouen, France; f Department of Urology, Hopital européen Georges Pompidou, Paris, France; g Department of Urology, CHU Tours, Tours, France; h Department of Urology, CHU Nîmes, Nîmes, France; i Department of Urology, Hopital Bicêtre, Paris, France; j Department of Urology, Hopital Henri Mondor, Créteil, France; k GRC 5 Predictive Onco-Uro, AP-HP, Urology, Pitie-Salpetriere Hospital, Sorbonne University, Paris, France; l Department of Urology, CH Mont-de-Marsan, Mont-de-Marsan, France; m Department of Urology, CHU Lille, Lille France; n Department of Urology, CHU Grenoble, Grenoble, France; o Department of Urology, Clinique Pasteur, Toulouse, France; p Department of Urology, Clinique Croix du Sud, Toulouse, France; q Department of Urology, Hopital Saint-Joseph, Paris France; r Department of Urology, CHU Poitiers, Poitiers, France; s Department of Urology, CHU Reims, Reims, France; t Department of Urology, Polyclinique Francheville, Périgueux, France; u Department of Urology, Hopital Tenon, Paris, France; v Department of Urology, CHU Clermont-Ferrand, Clermont-Ferrand, France; w Department of Urology, CHU Nice, Nice, France; x Department of Urology, CHU Rennes, Rennes, France; y Department of Urology, CHU Bordeaux, Bordeaux, France

**Keywords:** Renal cancer, Open conversion, Robot-assisted surgery, Laparoscopic surgery, Surgical complication, Intraoperative complication

## Abstract

**Background and objective:**

Data regarding open conversion (OC) during minimally invasive surgery (MIS) for renal tumors are reported from big databases, without precise description of the reason and management of OC. The objective of this study was to describe the rate, reasons, and perioperative outcomes of OC in a cohort of patients who underwent MIS for renal tumor initially. The secondary objective was to find the factors associated with OC.

**Methods:**

Between 2008 and 2022, of the 8566 patients included in the UroCCR project prospective database (NCT03293563), who underwent laparoscopic or robot-assisted minimally invasive partial (MIPN) or radical (MIRN) nephrectomy, 163 experienced OC. Each center was contacted to enlighten the context of OC: “emergency OC” implied an immediate life-threatening situation not reasonably manageable with MIS, otherwise “elective OC”. To evaluate the predictive factors of OC, a 2:1 paired cohort on the UroCCR database was used.

**Key findings and limitations:**

The incidence rate of OC was 1.9% for all cases of MIS, 2.9% for MIRN, and 1.4% for MIPN. OC procedures were mostly elective (82.2%). The main reason for OC was a failure to progress due to anatomical difficulties (42.9%). Five patients (3.1%) died within 90 d after surgery. Increased body mass index (BMI; odds ratio [OR]: 1.05, 95% confidence interval [CI]: 1.01–1.09, *p* = 0.009) and cT stage (OR: 2.22, 95% CI: 1.24–4.25, *p* = 0.008) were independent predictive factors of OC.

**Conclusions and clinical implications:**

In MIS for renal tumors, OC was a rare event (1.9%), caused by various situations, leading to impaired perioperative outcomes. Emergency OC occurred once every 300 procedures. Increased BMI and cT stage were independent predictive factors of OC.

**Patient summary:**

The incidence rate of open conversion (OC) in minimally invasive surgery for renal tumors is low. Only 20% of OC procedures occur in case of emergency, and others are caused by various situations. Increased body mass index and cT stage were independent predictive factors of OC.

## Take Home Message

The open conversion (OC) rate in minimally invasive surgery for renal tumors is low (1.9%). Only 20% of OC cases occur in a context of acute emergency; others are caused by various situations. Robot-assisted surgery may decrease the probability of OC.

## 1 Introduction

Minimally invasive surgery (MIS) for renal cancer is nowadays the standard of treatment for localized tumors, allowing the same oncological control of the disease with better perioperative outcomes than open surgery [1]. Nephron sparing surgery is preferred whenever it is judged feasible. MIS for partial nephrectomy (PN), especially robot-assisted PN, has increased worldwide, probably due to the facilities of robot-assisted surgery for the trickiest parts of the procedure (enucleation and parenchyma’s suture) [2]. When radical nephrectomy (RN) is preferred, MIS (laparoscopic or robot assisted) is still favored whenever possible [3].

Open conversion (OC) during MIS is a rare (2–10%) [4] and dreaded complication, especially with robot-assisted surgery when the surgeon is not directly operating on the patient. Since experience of surgeons is increasing regarding MIS [5], and maybe because experimented MIS surgeons feel more comfortable and accurate doing MIS rather than open surgery, OC rate is decreasing over time [4] and is lower in experimented centers [6].

Data regarding OC are reported from big national databases, without precise description of the reasons for OC [7], [8]. For instance, to the best of our knowledge, no data have ever been published distinguishing OC in a context of relative emergency (elective conversion) versus OC in a context of absolute emergency (emergency conversion). The observed predictive factors of OC were RN, older year of surgery, exclusive laparoscopic approach [9], and tumor complexity [7], [8], [10]. Logically, patients with OC have impaired postoperative outcomes with a higher rehospitalization rate [10].

The objective of the study was then to describe precisely the rate, reasons, and perioperative outcomes of OC in a modern cohort of patients who experienced MIS for renal tumors. The secondary objectives were to find predictive factors of OC.

## 2 Patients and methods

### 2.1 Study design

This was a retrospective study conducted in the framework of the UroCCR project (French network of research on kidney cancer, NCT03293563), which is a French multi-institutional prospectively maintained database of patients treated for kidney tumors. All patients received oral and written information about the objectives and methodology of the UroCCR project, and their written informed consent was obtained (CNIL authorization number DR-2013-206).

### 2.2 Precisions about surgeries

The selected patients were scheduled for the surgical treatment of renal masses with MIS (which encompassed laparoscopic or robot-assisted PN and RN) and had unplanned OC during the procedure. “Emergency OC” implied an immediate life-threatening situation not reasonably manageable with MIS (significant not repairable or precarious repaired active bleeding). We defined all other situations as “elective OC”.

### 2.3 Data measurements

Based on the UroCCR database, we evaluated the clinical characteristics of patients (age, sex, body mass index [BMI], previous abdominal surgery, size, side and complexity of tumor), surgeries, and postoperative outcomes. Each center was contacted to enlighten the context of OC (emergency or elective OC, patient repositioning, type of incision, surgeon’s experience, and call of the other senior surgeon) and describe the reason for OC precisely. Intraoperative complications were described using EAUiaiC classification [11], and postoperative complications were described using Clavien-Dindo classification [12].

### 2.4 Statistical analysis

Descriptive statistics included frequencies and proportions for categorical variables*.* Medians and interquartile ranges were reported for continuously coded variables. The statistical significance of differences in the medians and proportions was assessed with the Wilcoxon rank sum test, Pearson's chi-square test, and Fisher's exact test.

To evaluate the predictive factors of OC, a 2:1 paired cohort on the UroCCR database, matched on age, sex, and type of surgery, was used (*n* = 489). Matching was performed via propensity score matching (2:1, nearest neighbor matching with caliper 0.2). Within this cohort, uni- and multivariable logistic regression analyses (backward selection) were performed to evaluate potentially influencing factors on OC.

For all analyses, SPSS version 25.0 (IBM Corp., Armonk, NY, USA) and R software environment for statistical computing and graphics (version 3.4.3) was used. All tests were two sided, with a level of significance set at *p* < 0.05.

## 3 Results

### 3.1 OC rate and patients characteristics

From 2008 to 2022, of a total of 8566 registered MIS procedures in 25 centers, 163 were OC. The incidence rate of OC was 1.9% for all MIS procedures, ranged from 0.2% to 10.2% across centers, and was twice higher for minimally invasive RN (MIRN) than for minimally invasive PN (MIPN): 2.9% for MIRN and 1.4% for MIPN (Supplementary Table 1).

The included patients are summarized in Table 1: 71.2% were male and 39.3% had a BMI of >30 kg/m^2^. The median tumor size was 5.2 cm. The number of OC procedures performed was slightly more in right tumors (55.8%). Fifty-one patients (31.3%) had a history of abdominal surgery.

**Table 1:** Characteristics of patients

Characteristic	Value
Age, median (IQR)	66 (57–71)
Sex, *n* (%)	
Male	116 (71.2)
Female	47 (28.8)
Body mass index	
Median (IQR)	27.8 (24.6–32.3)
<30, *n* (%)	99 (60.7)
30–35, *n* (%)	30 (18.4)
>35, *n* (%)	26 (16.0)
No data, *n* (%)	8 (4.9)
ASA score, *n* (%)	
1	23 (14.1)
2	76 (46.6)
3–4	46 (28.2)
No data	18 (11.1)
Tumor characteristics	
Median size (IQR)	5.2 (3.5–8)
Size (cm), *n* (%)	
<4	46 (28.2)
4–7	45 (27.6)
>7	52 (31.9)
No data	20 (12.3)
Right side, *n* (%)	91 (55.8)
Left side, *n* (%)	72 (44.2)
Renal vein thrombus, *n* (%)	28 (17.2)
Cystic tumor, *n* (%)	22 (13.5)
Renal score, *n* (%)	
Low complexity	19 (11.6)
Intermediate complexity	44 (27.0)
High complexity	65 (39.9)
No data	35 (21.5)
Previous renal mass biopsy, *n* (%)	15 (9.2)
History of abdominal surgery, *n* (%)	51 (31.3)

ASA = American Society of Anesthesiologists; IQR = interquartile range.

### 3.2 Intra- and postoperative outcomes

OC concerned 82 (50.3%) MIPN and 81 (49.7%) MIRN cases; 64.6% of MIPN and 18.5% of MIRN cases were robot assisted (Table 2). OC procedures were mostly elective conversions (82.2%). Subcostal incision was the most frequent way to realize OC (69.3%). Dorsal decubitus repositioning was performed in 30 patients (18.4%), and call of the other senior surgeon occurred in only 36 patients (22.1%). The reasons for OC are precisely detailed in Table 3. The main reason for OC was a failure to progress due to anatomical difficulty (42.9%; mostly local tumor extension, intraperitoneal adherences, or toxic fat for MIPN). Unplanned conversion from PN to RN occurred in 19.5% of patients scheduled for MIPN.

**Table 2:** Characteristics of surgery

Characteristic	Value
Planned surgery	
Partial nephrectomy, *n* (%)	82 (50.3)
Robot assisted surgery	53/82 (64.6)
Laparoscopic surgery	15/82 (18.3)
Missing information regarding the use of a robot	12/82 (14.6)
Total nephrectomy, *n* (%)	81 (49.7)
Robot assisted surgery	15/81 (18.5)
Laparoscopic surgery	60/81 (74.1)
Missing information regarding the use of a robot	6/81 (7.4)
Approach, *n* (%)	
Retroperitoneal	15 (9.2)
Transperitoneal	148 (90.8)
Surgeon’s experience on mini-invasive renal surgery, *n* (%)	
<10	28 (17.2)
10–100	45 (27.6)
>100	81 (49.7)
No data	9 (5.5)
Box of open surgical tools, *n* (%)	
Already opened (systematically during laparoscopic surgery)	10 (6.1)
In the operating room, not opened during laparoscopic surgery	116 (71.2)
Not in the operating room	37 (22.7)
Dorsal decubitus repositioning during conversion, *n* (%)	30 (18.4)
Call of other senior surgeon, *n* (%)	36 (22.1)
Incision performed during conversion, *n* (%)	
Subcostal	113 (69.3)
Lumbotomy	38 (23.3)
Midline	5 (3.0)
Other (pararectus, bisubcostal, etc.)	4 (2.4)
Degree of emergency of open conversion, *n* (%)	
Absolute emergency (emergency conversion)	29 (17.8)
Relative emergency (elective conversion)	134 (82.2)
Reason of open conversion, *n* (%)	
Bleeding	42 (25.8)
Failure to progress due to anatomical difficulty	70 (42.9)
Failure to progress due to technical problems (insufflation)	2 (1.2)
Cancer control consideration	36 (22.1)
Accidental neighboring organ injury	13 (8.0)
Unplanned conversion from partial to radical nephrectomy, *n* (%)	
Yes	16 (9.8)
No	66 (40.5)
Not applicable (planned total nephrectomy)	81 (49.7)
Operative time, median [IQR] (extr)	180 [108–249] (45–529)
Intraoperative transfusion, *n* (%)	48 (29.4)
Estimated blood loss, median [IQR] (extr)	500 [250–1200] (0–4800)
Estimated blood loss ≥1000 ml, *n* (%)	51 (31.3)
Intraoperative complications (EAUiaiC classification), *n* (%)	
2	134 (82.2)
3	29 (17.8)
4	0 (0)
5	0 (0)
Length of stay (d), median [IQR] (extr)	6 [4–8] (2–53)
Postoperative complications, *n* (%)	
Postoperative transfusion	31 (19.0)
Urinary fistula	3 (1.8)
Abdominal hematoma	12 (7.4)
Wall hematoma	3 (1.8)
Wound abscess	4 (2.4)
Pseudoaneurysm	0 (0)
Ileus	4 (2.4)
Pancreatic fistula	2 (1.2)
Peritonitis	1 (0.6)
Urinary tract infection	2 (1.2)
Sepsis	6 (3.7)
Acute urinary retention	3 (1.8)
Clavien-Dindo IIIa	1 (0.6)
Clavien-Dindo IIIb	11 (6.7)
Clavien-Dindo IV	6 (3.7)
Clavien-Dindo V	5 (3.1)
Surgical reintervention, *n* (%)	11 (6.7)
Superficial abscess drainage	3 (1.8)
Upper tract drainage (JJ stent)	2 (1.2)
Renal vein tumor thrombectomy	1 (0.6)
Totalization (radical nephrectomy after initial partial nephrectomy)	2 (1.2)
Hemostasis	3 (1.8)
Hemostasis splenectomy	1 (0.6)
Peritonitis/washing peritoneum	1 (0.6)
Death, *n* (%)	5 (3.1)

extr = extreme values; IQR = interquartile range.

**Table 3:** Detailed reasons of open conversion

Characteristic	All patients (*n* = 163)	Planned partial nephrectomy (*n* = 82)	Planned radical nephrectomy (*n* = 81)
Bleeding, *n* (%)	42 (25.8)	22 (26.8)	20 (24.7)
Renal artery injury	7 (4.3)	5 (6.1)	2 (2.5)
Renal vein injury	9 (5.5)	3 (3.6)	6 (7.4)
Small vessel of renal pedicle	14 (8.6)	5 (6.1)	9 (11.1)
Vena cava	1 (0.6)	1 (1.2)	0 (0)
Aorta	1 (0.6)	1 (1.2)	0 (0)
Renal hyperpressure and diffuse bleeding after renal vein ligature (unexpected presence of accessory renal artery)	2 (1.2)	0 (0)	2 (2.5)
Ineffective clamping during partial nephrectomy	3 (1.8)	3 (3.6)	0 (0)
Active bleeding after unclamping during partial nephrectomy	4 (2.4)	4 (4.9)	0 (0)
Adrenal gland	1 (0.6)	0 (0)	1 (1.2)
Failure to progress due to anatomical difficulty, *n* (%)	70 (42.9)	32 (39.0)	38 (46.9)
Intraperitoneal adherences	20 (12.3)	9 (11.0)	11 (13.6)
Individualization of renal artery	6 (3.7)	3 (3.6)	3 (3.7)
Local tumor extension	28 (17.2)	4 (4.9)	23 (28.4)
Toxic fat	14 (8.6)	14 (17.1)	0 (0)
Upper pole access	1 (0.6)	1 (1.2)	0 (0)
Obesity/lack of space	1 (0.6)	1 (1.2)	0 (0)
Hepatomegaly	1 (0.6)	0 (0)	1 (1.2)
Failure to progress due to technical problems (insufflation), *n* (%)	2 (1.2)	2 (2.4)	0 (0)
Cancer control consideration, *n* (%)	36 (22.1)	21 (25.6)	15 (18.5)
Difficulty of tumor individualization	8 (4.9)	8 (9.8)	0 (0)
Tumor effraction/cystic rupture during tumorectomy	5 (3.1)	4 (4.9)	1 (1.2)
Doubt regarding positive surgical margin	5 (3.1)	5 (6.1)	0 (0)
Unexpected renal vein thrombus/uncontrollable renal vein thrombus	12 (7.3)	2 (2.4)	10 (12.3)
Unexpected vena cava thrombus	3 (1.8)	0 (0)	3 (3.7)
Unexpected retroperitoneal lymph nodes to remove	3 (1.8)	2 (2.4)	1 (1.2)
Accidental neighboring organ injury, *n* (%)	13 (8.0)	6 (6.1)	7 (8.6)
Superior mesenteric artery	1 (0.6)	0 (0)	1 (1.2)
Renal pelvis	1 (0.6)	1 (1.2)	0 (0)
Duodenal diverticulum	1 (0.6)	0 (0)	1 (1.2)
Liver	4 (2.4)	3 (3.6)	1 (1.2)
Pancreas	1 (0.6)	0 (0)	1 (1.2)
Spleen	2 (1.2)	1 (1.2)	1 (1.2)
Colon	3 (1.8)	1 (1.2)	2 (2.5)

Regarding intraoperative complications according to the EAUiaiC classification, the proportions of patients with grades 2 and 3 are the same as those of the patients with emergency and elective OC (18.8% and 82.2%, respectively). No patients had a grade 4 complication: one had a hemostatic splenectomy and another extensive intestine resection with stoma for ischemic colitis, but during subsequent surgeries. No patient had a grade complication 5 because there was no intraoperative death. However, five patients (3.1%) died within the 90 days after surgery: two because of multiple organ failure due to massive blood loss, two due to complications after peritonitis (anastomotic leakage after colectomy and duodenal diverticulum injury), and one after cardiorespiratory arrest due to severe aspiration pneumonia.

The median operative time and estimated blood loss (EBL) were 180 min (108–249) and 500 ml (250–1200; 0–4800), respectively. Fifty-one patients (31.3%) had >500 ml of EBL. The median length of stay was 6 days [4], [5], [6], [7], [8]. Postoperative complications are summarized in Table 2: there were 25 grade ≥3 complications, notably 11 patients (6.7%) with surgical reinterventions, mostly superficial abscess drainage and hemostasis.

More information regarding intra- and postoperative outcomes according to the degree of emergency of OC and the scheduled surgery is presented in Supplementary Tables 2 and 3. Emergency conversion was more associated with significant bleeding and unplanned conversion from PN to RN than elective conversion, but not with length of stay, surgical reintervention, or death (Supplementary Table 2). OC in case of planned RN occurred more frequently for novice surgeons (Supplementary Table 3).

Thirteen patients (8.0%) had a benign tumor and 13 (8.0%) had positive surgical margins (Supplementary Table 4).

### 3.3 Comparison between patients with and without OC (matched cohort n = 489)

In the matched-paired cohort, robot-assisted surgery was less frequent in the OC patients: 42% versus 67% without OC (77.1% vs 93.2% in case of MIPN and 19.5% vs 41.5% in case of MIRN). Patients with OC had a higher median tumor size (5.2 vs 4.5 cm), higher cT stage, and higher renal score. Patients with OC had logically worse perioperative outcomes than patients without OC (Table 4), with significantly longer length of stay, higher EBL, and more postoperative complications, surgical reinterventions, and death.

**Table 4:** Comparison between patients with or without unplanned open conversion, in a 2:1 paired cohort (matched on age, sex, and type of surgery)

Characteristic	Patients with open conversion (*n* = 163)	Patients with no conversion (*n* = 326)	*p* value
Age, median (IQR)	66 (57–71)	66 (57–71)	Paired on this criteria
Sex, *n* (%)			
Male	116 (71.2)	232 (71.2)	Paired on this criteria
Female	47 (28.8)	94 (28.8)	
Body mass index, median (IQR)	27.8 (24.6–32.3)	26.8 (23.6, 29.8)	0.009
ASA score, *n* (%)			
1–2	103 (63.2)	187 (57.4)	
3–4	48 (29.4)	100 (30.6)	0.5
No data	12 (7.4)	39 (12.0)	
Tumor characteristics			
Median size (IQR)	5.2 (3.5–8)	4.50 (3.00, 6.47)	0.019
cT stage, *n* (%)			
T1	89 (54.6)	205 (62.9)	0.006
T2	31 (19.0)	44 (13.5)	
T3	27 (16.6)	26 (8.0)	
No data	16 (9.8)	51 (15.6)	
Right side, *n* (%)	91 (55.8)	154 (47)	<0.001
Left side, *n* (%)	72 (44.2)	172 (53)	
Renal score, *n* (%)	9 (7, 10)	9 (6, 10)	0.023
Planned surgery, *n* (%)			
Partial nephrectomy	82 (50.3)	164 (50.3)	Paired on this criteria
Total nephrectomy	81 (49.7)	162 (49.7)	
Robotic-assisted surgery, *n* (%)	69 (42)	217 (67)	<0.001
Operative time, median (IQR)	200 (150, 240)	150 (113, 200)	<0.001
Estimated blood loss, median (IQR)	500 (250–1200)	100 (50–300)	<0.001
Postoperative nights, median [IQR] (extr)	6 (4–8)	3 (2, 4)	<0.001
Postoperative complications, *n* (%)			
Postoperative transfusion	31 (19.0)	7 (2.1)	<0.001
Urinary fistula	3 (1.8)	1 (0.3)	0.07
Abdominal hematoma	12 (7.4)	4 (1.2)	<0.001
Wall hematoma	3 (1.8)	1 (0.3)	0.07
Wound abscess	4 (2.4)	2 (0.6)	0.08
Pseudoaneurysm	0 (0)	0 (0)	–
Ileus	4 (2.4)	3 (0.9)	0.17
Pancreatic fistula	2 (1.2)	0 (0)	–
Peritonitis	1 (0.6)	0 (0)	–
Urinary tract infection	2 (1.2)	1 (0.3)	0.21
Sepsis	6 (3.7)	1 (0.3)	0.003
Acute urinary retention	3 (1.8)	7 (2.1)	0.8
Surgical reintervention, *n* (%)	11 (6.7)	3 (0.9)	<0.001
Superficial abscess drainage	3 (1.8)	0 (0)	0.01
Upper tract drainage (JJ stent)	2 (1.2)	1 (0.3)	0.21
Renal vein tumor thrombectomy	1 (0.6)	0 (0)	0.15
Totalization (total nephrectomy after initial partial nephrectomy)	2 (1.2)	0 (0)	0.04
Hemostasis	3 (1.8)	1 (0.3)	0.07
Hemostasis splenectomy	1 (0.6)	0 (0)	0.15
Peritonitis/washing peritoneum	1 (0.6)	0 (0)	0.15
Evisceration	0	1 (0.3)	0.47
Death, *n* (%)	5 (3.1)	1 (0.3)	0.02

ASA = American Society of Anesthesiologists; extr = extreme values; IQR = interquartile range.

### 3.4 Factors associated with OC

In univariable analyses (including BMI, American Society of Anesthesiologists score, tumor size, cT-stage, renal score, history of abdominal surgery, and robot assisted surgery) predicting OC, three factors reached independent predictor status: BMI (odds ratio [OR]: 1.06, 95% confidence interval [CI]: 1.02–1.09, *p* = 0.001), cT stage (OR: 2.23, 95% CI: 1.26–4.0, *p* = 0.006), and robotic assistance (OR: 0.44, 95% CI: 0.29–0.66, *p* = 0.0001). After including these in multivariable analyses, only BMI (OR: 1.05, 95% CI: 1.01–1.09, *p* = 0.009) and cT stage (OR: 2.22, 95% CI: 1.24–4.25, *p* = 0.008) reached independent predictor status; robotic assistance was close to statistical significance (OR: 0.66, 95% CI: 0.43–1.04, *p* = 0.0697).

## 4 Discussion

Based on our results, OC was a rare situation in MIS for renal tumors (1.9%). Fortunately, *emergency* OC was even more uncommon (about once every 300 procedures). However, surgeons have to prepare for this situation as concerned patients might have very serious complications and even die during or right after surgery. Our results might enlighten surgeons about various situations than can lead to an OC and help them anticipate this technically demanding surgery as best as they can.

Preoperative planning seems crucial to prevent technical difficulty during the procedure. Careful interpretation of a recent imaging, or even more three-dimensional reconstruction [13], [14] might help figure out anatomical boundaries of the tumor and better decide between nephron-sparing nephrectomy or RN, and laparoscopic or open surgery. Patient’s counseling should be adapted to each situation, detailing advantages, drawbacks, and risks of the planned surgery [15]. OC should always be discussed with the patient preoperatively and included in the “possibles” during informed consent. An elective OC during MIS due to concerns for oncological control or tumor extension should not be seen as a complication, but rather a judgment call by the surgeon to modify the surgical approach for better patient care.

In case of significant active bleeding, OC is not imperative, but appropriate experience in MIS and composure are mandatory to resolve the issue [16], as the surgeon has to be as fast and precise as possible for the vascular control (temporary clamping and effective stitches). Bed-side assistant has a crucial importance in case of robot-assisted surgery, since only he/she can apply clips or clean the surgical field with the suction device. After increasing the pneumoperitoneum, if OC is judged inevitable by the operator, at least bleeding should be decreased (application of accurate pressure with a tonsil swab or vascular clamp) during the critical period of OC. Nontechnical factors, such as communication [17], teamwork (good coordination with the anesthesiologist for hemorrhagic shock and quickly disposable blood products), and decision-making, are also critical to overcome such delicate situations. Call of the other senior surgeon could be particularly useful and was surprisingly underused in our cohort. Implementation of surgical simulation training programs addressing critical situations such as OC [18] could be interesting to be more armed in this exceptional situation, as processed in the aeronautic industry for the management of failures. If done more systematically during initial formation [19], and also periodically for confirmed surgeons, it might prevent inappropriate management of OC, facilitate communication, and increase decision-making.

Regarding intraoperative rates of OC, our results are slightly lower than previous data [4], [7], [8], [9]. This could be explained by a more recent cohort or more experienced surgeons and centers. Some OC might have been missed in our registry if any surgery had been registered falsely as an upfront open surgery in such situations. Another important result of our study is the first reported (to the best of our knowledge) rate of emergency OC: one out of 300 procedures. Furthermore, OC in MIS for renal tumors is more uncommon than in other nonurological surgeries [20], [21], attesting than MIS seems particularly appropriate for renal tumors and that urologists have long experience of MIS, especially robot-assisted surgery [22].

Our results are in accordance with previous studies regarding the predictive factors of OC such as obesity [4], nonrobotic approach [7], [9], and tumor stage [8]. Male sex as a predictive factor was also found by Klein et al [7], but we could not confirm it, since our paired cohort was matched on sex. These factors seem intuitive and underpin that the more difficult the surgery is expected to be, the higher would be the risk of OC. For the prevention of OC, surgeons should certainly take into consideration these factors; however, OC occurred in only 2% of MIS cases and would remain largely uncertain. Thus, intraoperative precautions, such as a large accessible surgical field with palpable anatomical landmarks or a box of open surgical tools in the operative room, could help facilitate OC and decrease its duration.

OC rate was almost twice higher for RN than for PN, and could be explained by a smaller proportion of robot-assisted surgeries during RN [9] and an underestimation of local tumor extension before surgery. In case of tumors planned for PN, unexpected local tumor extension might also be managed by conversion to RN, staying in MIS, without the necessity of OC. However, toxic fat leading to OC was an exclusive PN issue. There are several models that aim to predict adherent perinephric fat [23], [24], and their use should be encouraged. Nevertheless, the intraoperative difficulties caused by toxic fat, leading in worst cases to OC, are very hard to anticipate, and widely depend on surgeon’s experience and tumor location.

Our study has several limitations: first, detailed reasons and modalities of OC were assessed retrospectively based on operative reports, leading to potential information biases. Second, OC rate could have been underestimated if a surgery had been registered falsely as an upfront open surgery in our multicenter database. Third, the only way to find the predictive factors of OC was to create a paired cohort on the UroCCR database, matched on age, size of tumor, and type of surgery because two groups would really be unbalanced using all the UroCCR patients (thousands of patients). This could have led to a lack of power to detect the predictive factors of OC.

## 5 Conclusions

OC was a rare situation in MIS for renal tumors (1.9%), and emergency OC occurred once in every 300 procedures. Increased BMI and cT stage were independent patient-related predictive factors of OC. Patients concerned by OC might have very serious complications and even die during or right after surgery. Our results might enlighten surgeons about various situations than can lead to OC, and help them anticipate this event as best as they can.

  ***Author contributions*:** Nicolas Branger had full access to all the data in the study and takes responsibility for the integrity of the data and the accuracy of the data analysis.

  *Study concept and design*: Branger.

*Acquisition of data*: All authors.

*Analysis and interpretation of data*: Branger, Knipper.

*Drafting of the manuscript*: Branger, Pignot, Bernhard.

*Critical revision of the manuscript for important intellectual content*: Branger, Knipper, Bernhard.

*Statistical analysis*: Knipper.

*Obtaining funding*: None.

*Administrative, technical, or material support*: Bernhard.

*Supervision*: Branger, Bernhard.

*Other*: None.

  ***Financial disclosures***: Nicolas Branger certifies that all conflicts of interest, including specific financial interests and relationships and affiliations relevant to the subject matter or materials discussed in the manuscript (eg, employment/affiliation, grants or funding, consultancies, honoraria, stock ownership or options, expert testimony, royalties, or patents filed, received, or pending), are the following: None.

  ***Funding/Support and role of the sponsor*:** None.

  ***Acknowledgments*:** The authors wish to thank the entire team of the UroCCR network (residents, clinicians and local clinical research associates in each center, and dedicated clinical research associates of UroCCR in Bordeaux), especially Guillaume Herman for the coordination and reminders sent to all centers, and Arna Geshkovska for the help and remarks in statistics.

## References

[1] Bedke J., Albiges L., Capitanio U. (2021). Updated European Association of Urology guidelines on renal cell carcinoma: nivolumab plus cabozantinib joins immune checkpoint inhibition combination therapies for treatment-naïve metastatic clear-cell renal cell carcinoma. Eur Urol.

[2] Alameddine M., Koru-Sengul T., Moore K.J. (2019). Trends in utilization of robotic and open partial nephrectomy for management of cT1 renal masses. Eur Urol Focus.

[3] Gershman B., Bukavina L., Chen Z. (2020). The Association of robot-assisted versus pure laparoscopic radical nephrectomy with perioperative outcomes and hospital costs. Eur Urol Focus.

[4] Luzzago S., Rosiello G., Pecoraro A. (2021). Contemporary rates and predictors of open conversion during minimally invasive partial nephrectomy for kidney cancer. Surg Oncol.

[5] Paulucci D.J., Abaza R., Eun D.D., Hemal A.K., Badani K.K. (2017). Robot-assisted partial nephrectomy: continued refinement of outcomes beyond the initial learning curve. BJU Int.

[6] Xia L., Pulido J.E., Chelluri R.R. (2018). Hospital volume and outcomes of robot-assisted partial nephrectomy. BJU Int.

[7] Klein G., Wang H., Elshabrawy A. (2021). Analyzing National incidences and predictors of open conversion during minimally invasive partial nephrectomy for cT1 renal masses. J Endourol.

[8] Roberts J.L., May A., Hamilton Z. (2021). Unplanned open conversion during radical or partial nephrectomy: comparing outcomes and trends. Urology.

[9] Khanna A., Campbell S.C., Murthy P.B., Ericson K.J., Nyame Y.A., Abouassaly R. (2020). Unplanned conversion from minimally invasive to open kidney surgery: the impact of robotics. J Endourol.

[10] Razdan S., Okhawere K., Wilson M. (2022). Conversion to open radical or partial nephrectomy associated with unplanned hospital readmission after attempted minimally invasive approach. J Laparoendosc Adv Surg Tech.

[11] Biyani C.S., Pecanka J., Rouprêt M., Jensen J.B., Mitropoulos D. (2020). Intraoperative Adverse Incident Classification (EAUiaiC) by the European Association of Urology ad hoc Complications Guidelines Panel. Eur Urol.

[12] Clavien P.A., Barkun J., de Oliveira M.L. (2009). The Clavien-Dindo classification of surgical complications: five-year experience. Ann Surg.

[13] Michiels C., Khene Z.E., Prudhomme T. (2023). 3D-Image guided robotic-assisted partial nephrectomy: a multi-institutional propensity score-matched analysis (UroCCR study 51). World J Urol.

[14] Piramide F., Kowalewski K.F., Cacciamani G. (2022). Three-dimensional model–assisted minimally invasive partial nephrectomy: a systematic review with meta-analysis of comparative studies. Eur Urol Oncol.

[15] Azhar R.A., Bochner B., Catto J. (2016). Enhanced recovery after urological surgery: a contemporary systematic review of outcomes, key elements, and research needs. Eur Urol.

[16] Reeves F., George N., Challacombe B. (2023). Red out: bleeding during robotic retroperitoneal lymph node dissection and strategies to manage it. Eur Urol Open Sci.

[17] Lingard L., Regehr G., Orser B. (2008). Evaluation of a preoperative checklist and team briefing among surgeons, nurses, and anesthesiologists to reduce failures in communication. Arch Surg.

[18] Graafland M., Schraagen J.M., Schijven M.P. (2012). Systematic review of serious games for medical education and surgical skills training. Br J Surg.

[19] Wang R.S., Ambani S.N. (2021). Robotic surgery training: current trends and future directions. Urol Clin.

[20] Gheza F., Esposito S., Gruessner S., Mangano A., Fernandes E., Giulianotti P.C. (2019). Reasons for open conversion in robotic liver surgery: a systematic review with pooled analysis of more than 1000 patients. Int J Med Robot.

[21] Beane J.D., Pitt H.A., Dolejs S.C., Hogg M.E., Zeh H.J., Zureikat A.H. (2018). Assessing the impact of conversion on outcomes of minimally invasive distal pancreatectomy and pancreatoduodenectomy. HPB.

[22] Chung G., Hinoul P., Coplan P., Yoo A. (2021). Trends in the diffusion of robotic surgery in prostate, uterus, and colorectal procedures: a retrospective population-based study. J Robot Surg.

[23] Davidiuk A.J., Parker A.S., Thomas C.S. (2014). Mayo adhesive probability score: an accurate image-based scoring system to predict adherent perinephric fat in partial nephrectomy. Eur Urol.

[24] Borregales L.D., Adibi M., Thomas A.Z. (2021). Predicting adherent perinephric fat using preoperative clinical and radiological factors in patients undergoing partial nephrectomy. Eur Urol Focus.

