# Supplementary material for: Preparing for the Worst: Management and Predictive Factors of Open Conversion During Minimally Invasive Renal Tumor Surgery (UroCCR-135 Study)
Source: Eur Urol Open Sci. 2024 Mar 28;63:89–95. doi: 10.1016/j.euros.2024.03.009 (PMC10997889; doi:10.1016/j.euros.2024.03.009)
Supplement: Supplementary data 1 [file mmc1.docx]

Supplementary Table 1: Repartition of rates of open conversion between centers, during minimally invasive renal surgery (MIRS), minimally invasive partial nephrectomy (MIPN) and minimally invasive total nephrectomy (MITN)

| Center number | Total number of open conversion during MIRS | open conversion during planned MIPN | open conversion during planned MITN | Number of MIRS | Number of MIPN | Number of MITN | Global open conversion rate during MIRS | Global open conversion rate during MIPN | Global open conversion rate during MITN |
| --- | --- | --- | --- | --- | --- | --- | --- | --- | --- |
| 1 | 17 | 13 | 4 | 1979 | 1315 | 664 | 0,9% | 1,0% | 0,6% |
| 2 | 33 | 15 | 18 | 771 | 557 | 214 | 4,3% | 2,7% | 8,4% |
| 3 | 13 | 4 | 9 | 618 | 350 | 268 | 2,1% | 1,1% | 3,4% |
| 4 | 16 | 4 | 12 | 600 | 400 | 200 | 2,7% | 1,0% | 6,0% |
| 5 | 6 | 4 | 2 | 539 | 382 | 157 | 1,1% | 1,0% | 1,3% |
| 6 | 1 | 1 | 0 | 537 | 377 | 160 | 0,2% | 0,3% | 0,0% |
| 7 | 3 | 3 | 0 | 506 | 407 | 99 | 0,6% | 0,7% | 0,0% |
| 8 | 7 | 5 | 2 | 385 | 256 | 129 | 1,8% | 2,0% | 1,6% |
| 9 | 10 | 7 | 3 | 315 | 243 | 72 | 3,2% | 2,9% | 4,2% |
| 10 | 10 | 6 | 4 | 298 | 197 | 101 | 3,4% | 3,0% | 4,0% |
| 11 | 6 | 4 | 2 | 261 | 197 | 64 | 2,3% | 2,0% | 3,1% |
| 12 | 14 | 2 | 12 | 231 | 85 | 146 | 6,1% | 2,4% | 8,2% |
| 13 | 1 | 1 | 0 | 228 | 190 | 38 | 0,4% | 0,5% | 0,0% |
| 14 | 4 | 0 | 4 | 214 | 158 | 56 | 1,9% | 0,0% | 7,1% |
| 15 | 2 | 0 | 2 | 213 | 154 | 59 | 0,9% | 0,0% | 3,4% |
| 16 | 6 | 4 | 2 | 188 | 130 | 58 | 3,2% | 3,1% | 3,4% |
| 17 | 2 | 0 | 2 | 137 | 72 | 65 | 1,5% | 0,0% | 3,1% |
| 18 | 1 | 0 | 1 | 110 | 79 | 31 | 0,9% | 0,0% | 3,2% |
| 19 | 1 | 0 | 1 | 101 | 49 | 52 | 1,0% | 0,0% | 1,9% |
| 20 | 2 | 2 | 0 | 80 | 59 | 21 | 2,5% | 3,4% | 0,0% |
| 21 | 2 | 1 | 1 | 80 | 50 | 30 | 2,5% | 2,0% | 3,3% |
| 22 | 1 | 0 | 1 | 58 | 6 | 52 | 1,7% | 0,0% | 1,9% |
| 23 | 1 | 1 | 0 | 57 | 36 | 21 | 1,8% | 2,8% | 0,0% |
| 24 | 2 | 2 | 0 | 41 | 13 | 28 | 4,9% | 15,4% | 0,0% |
| 25 | 2 | 2 | 0 | 19 | 15 | 4 | 10,5% | 13,3% | 0,0% |
|  |  |  |  |  |  |  |  |  |  |
| **TOTAL** | **163** | **81** | **82** | **8566** | **5777** | **2789** | **1,9%** | **1,4%** | **2,9%** |
|  |  |  |  |  |  |  |  |  |  |

Supplementary Table 2: Comparision of outcomes between patients with elective or emergency conversion

| Characteristic | Elective conversion (n=134) | Emergency conversion  (n=29) | p |
| --- | --- | --- | --- |
| Planned surgery  Partial nephrectomy  Total nephrectomy | 66 (49%)  68 (51%) | 16 (55%)  13 (45%) | 0.6 |
| Median size [IQR]  Size  < 4cm  4-7cm  > 7cm  No data  Right side  Left side  Renal score (median (IQR))  Low complexity  Intermediate complexity  High complexity  No data | 5.20 (3.50, 8.00)  38 (28%)  37 (28%)  41 (31%)  18 (13%)  75 (56%)  59 (44%)  9 (7, 10)  16 (12%)  35 (26%)  54 (40%)  21 (22%) | 5.50 (3.50, 8.00)  8 (28%)  8 (28%)  11 (38%)  2 (6.9%)  16 (55%)  13 (45%)  9 (7, 10)  3 (10%)  9 (31%)  11 (38%)  6 (21%) | 0.7  0.8  0.9  0.9 |
| Surgeon’s experience on mini-invasive renal surgery  < 10  10-100  > 100  No data | 23 (17%)  37 (28%)  68 (51%)  6 (4.5%) | 5 (17%)  8 (28%)  13 (45%)  3 (10%) | 0.6 |
| Dorsal decubitus repositioning during conversion | 29 (22%) | 1 (3.4%) | 0.009 |
| Call of other senior surgeon | 31 (23%) | 5 (17%) | 0.8 |
| Incision performed during conversion  Subcostal  Lumbotomy  Midline  Other (Pararectus, bisubcostal…) | 90 (67%)  33 (25%)  4 (3.0%)  4 (3.0%) | 23 (79%)  5 (17%)  1 (3.4%)  4 (3.0%) | 0.8 |
| Reason of open conversion  Bleeding  Failure to progress due to anatomical difficulty  Failure to progress due to technical problems (insufflation)  Cancer control consideration  Accidental neighbouring organs injury | 15 (11.1%)  70 (52%)  2 (1.5%)  36 (27%)  11 (8.2%) | 27 (93%)  0  0  0  2 (6.9%) | < 0.001 |
| Unplanned Conversion From Partial to Radical Nephrectomy  Yes  No  Not applicable (planned total nephrectomy) | 7 (5.2%)  59 (44%)  68 (51%) | 8 (28%)  8 (28%)  13 (45%) | 0.002 |
| Median operative time median [IQR] | 203 (150, 240) | 180 (150, 239) | 0.8 |
| Intraoperative transfusion | 30 (22%) | 18 (62%) | < 0.001 |
| Estimated blood loss median (IQR)  Estimated blood loss ≥ 1000 mL | 460 (200, 1,000)  31 (23.1%) | 1,650 (750, 2,000)  20 (70%) | < 0.001  < 0.001 |
| Intraoperative complications (EAUiaiC classification)  2  3  4  5 | 132 (99%)  0 (0%)  2 (1.5%)  0 (0%) | 0 (0%)  29 (100%)  0 (0%)  0 (0%) | < 0.001 |
| Postoperative nights median [IQR] (extr) | 6 (4, 8) | 5 (4, 8) | 0.9 |
| Postoperative complications  Postoperative transfusion  Urinary fistula  Abdominal hematoma  Wall hematoma  Wound abcess  Pseudoaneurysm  Ileus  Pancreatic fistula  Peritonitis  Urinary tract infection  Sepsis  Acute urinary retention | 21 (16%)  2 (1.5%)  12 (9.0%)  2 (1.5%)  3 (2.2%)  0 (0%)  4 (3.0%)  2 (1.5%)  1 (0.7%)  2 (1.5%)  6 (4.5%)  3 (2.2%) | 10 (34%)  1 (3.4%)  0 (0%)  1 (3.4%)  1 (3.4%)  0 (0%)  0 (0%)  0 (0%)  0 (0%)  0 (0%)  0 (0%)  0 (0%) | 0.02  ns  -  ns  ns  -  -  -  -  -  -  - |
| Surgical reintervention  Superficial abcess drainage  Upper tract drainage (JJ stent)  Renal vein tumor thrombectomy  Totalisation (total nephrectomy after initial partial nephrectomy)  Hemostasis  Hemostastasis splenectomy  Peritonitis / washing peritoneum | 10 (7.5%)  2 (1.5%)  1 (0.7%)  1 (0.7%)  2 (1.2%)  3 (1.8%)  1 (0.6%)  1 (0.6%) | 1 (3.4%)  0 (0%)  1 (0.7%)  0 (0%)  0 (0%)  0 (0%)  0 (0%)  0 (0%) | 0.8  -  ns  -  -  -  -  - |
| Death | 4 (3.0%) | 1 (3.4%) | 0.6 |

Supplementary Table 3: Comparision of outcomes between patients with partial or radical nephrectomy

| Characteristic | Planned partial nephrectomy (n=82) | Planned radical nephrectomy  (n=81) | p |
| --- | --- | --- | --- |
| Median size (IQR)  Size  < 4cm  4-7cm  > 7cm  No data  Right side  Left side  Renal score, median (IQR)  Low complexity  Intermediate complexity  High complexity  No data | 4.00 (3.00, 5.50)  35 (43%)  26 (32%)  10 (12%)  11 (13%)  57 (70%)  25 (30%)  8 (6, 9)  14 (17%)  30 (37%)  16 (20%)  22 (27%) | 7.55 (5.00, 9.00)  11 (14%)  19 (23%)  42 (52%)  9 (11%)  34 (42%)  47 (58%)  10 (9, 10)  5 (6.2%)  14 (17%)  49 (60%)  13 (16%) | <0.001  <0.001  <0.001 |
| Surgeon’s experience on mini-invasive renal surgery  < 10  10-100  > 100  No data | 9 (11%)  18 (22%)  52 (63%)  3 (3.7%) | 19 (23%)  27 (33%)  29 (36%)  6 (7.4%) | 0.004 |
| Dorsal decubitus repositioning during conversion | 11 (13%) | 19 (23%) | 0.001 |
| Call of other senior surgeon | 16 (20%) | 20 (25%) | 0.2 |
| Type of conversion, n (%)  elective conversion  emergency conversion | 66 (80%)  16 (20%) | 68 (84%)  13 (16%) | 0.6 |
| Incision performed during conversion  Subcostal  Lumbotomy  Midline  Other (Pararectus, bisubcostal…) | 59 (72%)  21 (26%)  0 (0%)  1 (1.2%) | 54 (67%)  17 (21%)  5 (6.2%)  3 (3.7%) | 0.11 |
| Reason of open conversion  Bleeding  Failure to progress due to anatomical difficulty  Failure to progress due to technical problems (insufflation)  Cancer control consideration  Accidental neighbouring organs injury | 22 (27%)  31 (38%)  2 (2.4%)  21 (26%)  6 (7.3%) | 20 (25%)  39 (48%)  0 (0%)  15 (19%)  7 (8.6%) | ns  ns  -  ns  ns |
| Unplanned Conversion From Partial to Radical Nephrectomy  Yes  Not applicable (planned total nephrectomy) | 15 (18%)  0 (0%) | 0 (0%)  81 (100%) | - |
| Median operative time median [IQR] | 190 (152, 240) | 204 (143, 238) | 0.6 |
| Intraoperative transfusion | 20 (24%) | 28 (35%) | 0.15 |
| Estimated blood loss median [IQR] (extr) | 500 (200, 1075) | 600 (300, 1200) | 0.3 |
| Intraoperative complications (EAUiaiC classification)  2  3  4  5 | 66 (80%)  16 (20%)  0 (0%)  0 (0%) | 66 (81%)  13 (16%)  2 (2.5%)  0 (0%) | 0.4 |
| Postoperative nights median [IQR] (extr) | 6 (4, 9) | 6 (4, 8) | 0.12 |
| Postoperative complications  Postoperative transfusion  Urinary fistula  Abdominal hematoma  Wall hematoma  Wound abcess  Pseudoaneurysm  Ileus  Pancreatic fistula  Peritonitis  Urinary tract infection  Sepsis  Acute urinary retention | 13 (16%)  3 (3.7%)  8 (9.8%)  2 (2.4%)  0 (0%)  0 (0%)  2 (2.4%)  2 (2.4%)  1 (1.2%)  0 (0%)  2 (2.4%)  0 (0%) | 18 (22.2%)  0 (0%)  4 (4.9%)  1 (1.2%)  4 (4.9%)  0 (0%)  2 (2.5%)  0 (0%)  0 (0%)  2 (2.5%)  4 (4.9%)  3 (3.7%) | ns  -  ns  ns  -  -  ns  -  -  -  ns  - |
| Surgical reintervention  Superficial abcess drainage  Upper tract drainage (JJ stent)  Renal vein tumor thrombectomy  Totalisation (total nephrectomy after initial partial nephrectomy)  Hemostasis  Hemostastasis splenectomy  Peritonitis / washing peritoneum | 5 (6.1%)  0 (0%)  2 (2.4%)  0 (0%)  2 (2.4%)  1 (1.2%)  0 (0%)  0 (0%) | 6 (7.4%)  2 (2.5%)  0 (0%)  1 (1.2%)  0 (0%)  2 (2.5%)  1 (1.2%)  1 (1.2%) | ns  -  -  -  -  ns  -  - |
| Death | 1 (1.2%) | 4 (4.9%) | ns |

Supplementary Table 4 Pathological results

| Characteristic | Value |
| --- | --- |
| Tumor stage  T1a  T1b  T2  T3a  T3b  T3c  T4  Tx / no data | 35 (21.5%)  24 (14.7%)  10 (6.1%)  67 (41.1%)  4 (2.4%)  1 (0.6%)  3 (1.8%)  19 (11.7%) |
| Node stage  N0 / Nx  N1/ N2 | 161 (98.8%)  2 (1.2%) |
| Histologic subtype  Malignant  Clear cell RCC  Papillary RCC  Chromophobe RCC  Other  Benign  Oncocytoma  Angiomyolipoma  Simple cyst  Other  No data | 144 (88.3%)  118 (72.4%)  12 (7.4%)  6 (3.7%)  8 (4.9%)  13 (8.0%)  7 (4.3%)  1 (0.6%)  1 (0.6%)  4 (2.5%)  6 (3.7%) |
| Grade  1  2  3  4  NR | 9 (5.5%)  52 (31.9%)  48 (29.5%)  25 (15.3%)  29 (17.8%) |
| Margins  R0  R1  No data | 127 (77.9%)  13 (8.0%)  24 (14.7%) |
